# Highly Dual Antifouling and Antibacterial Ultrafiltration Membranes Modified with Silane Coupling Agent and Capsaicin-Mimic Moieties

**DOI:** 10.3390/polym12020412

**Published:** 2020-02-11

**Authors:** Lili Zhang, Yuanyuan Tang, Xiaohui Jiang, Liangmin Yu, Changyun Wang

**Affiliations:** 1 Key Laboratory of Marine Chemistry Theory and Technology, Ministry of Education, Ocean University of China, Qingdao 266100, China; zllouc@163.com (L.Z.); tyy_2013@yeah.net (Y.T.); tchb666@163.com (X.J.); 2 Open Studio for Marine Corrosion and Protection, Pilot National Laboratory for Marine Science and Technology, Qingdao 266100, China; 3 Key Laboratory of Marine Drugs, The Ministry of Education of China, School of Medicine and Pharmacy, Ocean University of China, Qingdao 266100, China; changyun@ouc.edu.cn

**Keywords:** ultrafiltration, capsaicin-mimic, silane coupling agent, antibacterial, antifouling

## Abstract

Dual antifouling and antibacterial polysulfone(PSf)/polyethersulfone(PES) hybrid membranes were developed by the synergy of capsaicin-mimic *N*-(5-methyl acrylamide-2,3,4 hydroxy benzyl) acrylamide (AMTHBA) and vinyl triethylene (b-methoxy ethoxy) silane (VTMES). First, AMTHBA as a natural antimicrobial agent was incorporated into a casting solution via “microwave-assistance (MWA) in situ polymerization-blending” process to construct a hydroxyl-rich environment. Then, VTMES crosslinked to a hydroxyl-rich polymer matrix via hydrolytic condensation, and the influence of VTMES content on the hybrid membrane properties was systematically investigated. When the VTMES added amount was 1.0 wt %, the hybrid membrane achieved an optimal separation performance including a steady-state humic acid (HA) (5 mg/L) permeation flux of 326 L·m^−2^·h^−1^ and a rejection percentage of 97%. The antibacterial tests revealed that the hybrid membranes exhibited sustained bactericidal activity and effective inhibition of bacterial adhesion. Besides, the dual-functional membranes were clean as new after two-cycles filtration (with a cleaning efficiency of ~90%), indicating that the network silicone film on the surface benefits the foulant repellence. Hopefully, the dual-functional membranes constructed in this study can be applicable to the pretreatment stage of water treatment.

## 1. Introduction

Ultrafiltration (UF), as a sustainable cost-effective low-pressure wastewater treatment technology, yielding good removal of turbidity and microbes, has been extensively applied in water purification and reclamation [1,2,3,4]. However, membrane fouling, particularly biological fouling and organic fouling, begins, once the membrane is exposed to a complex watery environment [5,6,7]. Moreover, many issues (decrease in filtration efficiency, increase in the energy consumption, deterioration in water quality, acceleration in membrane aging, etc.) are brought out with the adhesion and accumulation of organic, biological and colloidal substances on the membrane surface or even inside membrane pores [8,9]. Worst still, it is proverbially difficult to prevent or eliminate. To solve the abovementioned problems, various antifouling membranes have been designed to enhance antifouling and antibacterial properties ever since the application of membrane separation technology [10,11,12].

According to different ingredients and the corresponding function mechanisms, antifouling membranes can be divided into three strategies: (i) hydrophilic membranes for preventing the initial attachment of organic fouling and bacteria; (ii) bactericidal membranes for killing attached bacteria and preventing the formation of viable biofilm, which is principally against biofouling; and (iii) dual-functional bactericidal membranes for reducing both organic fouling and biofouling [13,14,15,16]. Among these, dual-functional bactericidal modification combining two strategies into one system is considered an ideal method. For example, Zhang et al. [17] designed a dual-functional antibacterial surface with a two-step surface modification strategy, aiming to acquire the properties of fouling resistance and antibacterial performance. Besides, Xie et al. [18] co-deposited polydopamine (PDA) and a zwitterionic polymer onto a polyethersulfone (PES) substrate and then immersed the surface in a AgNO_3_ solution, leading to satisfactory antibacterial and antibiofouling properties. More recently, Wu et al. [19] synthesized a bifunctional membrane by incorporating silver–polydopamine nanohybrid (Ag–PDA) nanocomposites in a polysulfone membrane matrix. These results indicated the obvious advantages of dual functionality. However, due to the absence of strong bonding with surrounding polymers, active particles can be gradually leached out during operation, which could deteriorate the membrane antifouling efficiency [20]. In addition, the release of additional agents may have some potential impacts on the environment and human health [21].

Capsaicin-mimic materials used as natural antifungal and antibacterial agents exhibit strong inhibition effects on various microbes and have been widely applied to resist marine or biomedical bacterial attachment [22,23,24,25]. Numerous studies have reported capsaicin and its derivatives are in urgent demand to meet the increasing challenges in the fabrication of bactericidal membranes [26,27]. In our previous research, we used strategies (i) and (ii) and incorporated *N*-(5-methyl acrylamide-2,3,4 hydroxy benzyl) acrylamide (AMTHBA) in a membrane matrix, which resulted in excellent hydrophilicity and antibacterial [28]. Although modified surfaces containing capsaicin-mimic moieties can kill the attached bacteria, the residual dead bacteria will still contaminate the membrane, which may cause immune responses or inflammation [29,30]. Therefore, it is imperative to develop a novel membrane by introducing antifouling capabilities to antibacterial surfaces to achieve dual functions (antifouling and antibacterial).

Recently, silane coupling agents have attracted a great deal of interest for their simplicity, versatility, and wide applicability [31,32]. It has been reported that silane coupling agents show excellent mechanical durability and antifouling property due to their special chemical structures, which can react with both inorganic fillers and organic substrates, and have been widely used to resist dust attachment and increase tensile moduli of composite materials in coatings industry [33,34]. Inspired by the antifouling properties of silane coupling agents, we anticipate that the co-modification of silane coupling agents and capsaicin-mimic moieties can endow polymeric membranes with antifouling and antibacterial properties.

In this work, a two-step modification strategy was used to prepare a dual-functional polymer membrane. A polysulfone(PSf)/PES substrate was selected as a polymeric substrate. First, AMTHBA was incorporated into the PSf/PES-based casting solution via a “microwave-assistance (MWA) in situ polymerization-blending” process, since its plentiful reactive hydroxyl groups could be used to crosslink with hydrolyzed silane coupling agents. Then, vinyl triethylene (b-methoxy ethoxy) silane (VTMES) was added to the AMTHBA-containing casting solution and heated with MWA. The successful incorporation of VTMES into the membrane matrix was confirmed by attenuated total reflection Fourier transform infrared spectroscopy (FTIR/ATR) and energy-dispersive spectroscopy (EDS). The antibacterial properties of the modified membranes were demonstrated via sterilization ratio and fluorescence microscopy observation. The antifouling properties of the membrane were evaluated by cycle-dependent filtration experiments employing a humic acid (HA) solution (5 mg/L) as a model organic foulant. The results verified that the dual-functional membrane functionalization with AMTHBA and VTMES is a promising strategy to prevent membrane fouling in water treatment.

## 2. Materials and Methods

### 2.1. Materials

**Figure 1:**
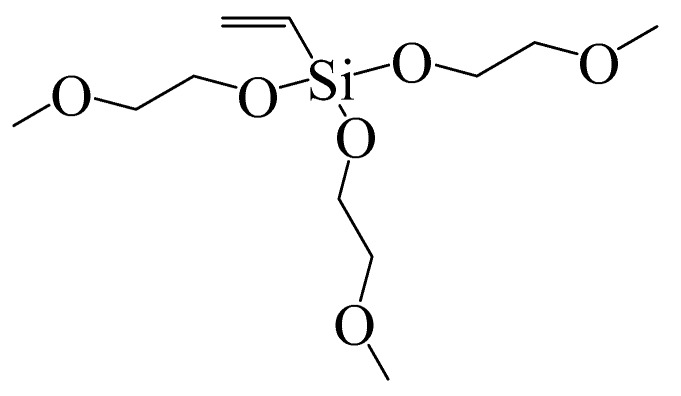
Molecular structure of vinyl triethylene (b-methoxy ethoxy) silane (VTMES).

PSf (Ultrason E2020P) and PES (Ultrason S6010) were supplied by BASF (Ludwigshafen, Germany). AMTHBA was made by our laboratory, and the details of generating it were reported in our previous work [28]. VTMES (Figure 1) was acquired from Dow chemical Co. Ltd. (Shanghai, China). *N*,*N*-dimethyl acetamide (DMAc, 99.0%) and polyethylene glycol (PEG, *M*_w_ = 380–430 Da) were purchased from Sinopharm Chemical Reagent Co. Ltd. (Shanghai, China). 2,2′-Azodiisobu-tyronitrile (AIBN, AR) was obtained from Tianjin Damao Chemical Reagents (Tianjin, China). Bovine serum albumin (BSA, *M*_w_ = 67,000 Da) and HA were acquired from Sigma-Aldrich (Burlington, MA, USA). All the chemical reagents were used as received without further purification.

### 2.2. Membrane Preparation

**Figure 2:**
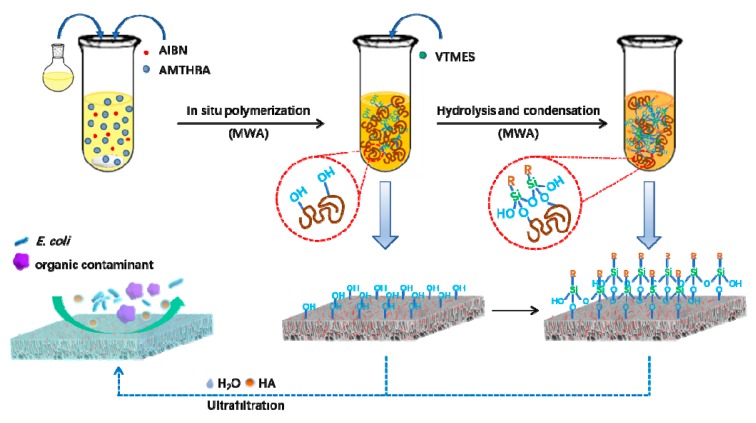
Schematic illustration for the fabrication of dual antifouling and antibacterial hybrid membranes.

The schematic illustration for the fabrication of hybrid membranes is displayed in Figure 2. All membranes were prepared with the L-S phase inversion method, which is widely used in the application of membrane separation [35,36]. Firstly, the homogenous solution of PSf/PES was prepared in DMAc as a solvent, and PEG-400 acted as a pore-forming agent by magnetic stirring at 50 °C for 10 h. AMTHBA and AIBN were dissolved in DMAc through ultrasonication for 1 min to obtain a stable solution, and the dosage was determined according to the previous report [28]. Then, a certain amount (2 wt %) of AMTHBA in DMAc was added to the PSf/PES solution and reacted via microwave irradiation at 90 °C for 10 min. Finally, different amounts of VTMES (0.5, 1.0, 2.0, and 3.0 wt %) were added slowly into the solution under constant stirring to obtain homogeneous casting solutions, and these membranes were designed as PSf/PES-AM-VT 0.5, PSf/PES-AM-VT 1.0, PSf/PES-AM-VT 2.0, and PSf/PES-AM-VT 3.0, respectively. The compositions of various membranes were shown in Table 1. After being vacuum degassed, the homogeneous solutions were poured on a clean glass plate and casted by a film applicator, and then a thin film was acquired after immersing into a deionized water (DI water) coagulation bath at 30 °C. The as-prepared membranes were rinsed several times and stored in DI water at room temperature for further characterization and evaluation.

### 2.3. Membrane Characterization

Surface chemical characterizations of the neat and modified polymer membranes were investigated by FTIR/ATR (TENSOR 27, Broker, Germany). A scanning electron microscope (SEM, S-4800, Hitachi, Japan) was employed to observe the morphologies of the membranes. A three-dimenstional (3D) measuring laser microscope (LEXT, OLS4000, Olympus, Japan) was employed to acquire the soiled surface morphologies of the membranes after UF experiments. The mechanical properties of the membranes were evaluated with a tensile testing equipment (AGS-J, China). The static and dynamic water contact angles (WCAs) were measured by a DropMeter contact angle measuring system (DSA100, Kruss, Hamburg, Germany) at room temperature. A UV–VIS spectrophotometer (TU-1901, Pgeneral, Beijing, China) was applied to determine the absorbance of feed and permeate solutions.

### 2.4. Filtration Experiments

The permeation and separation properties of the neat and modified membranes were evaluated using a commercial cross-flow filtration setup (effective area = 8.3 cm^2^) supplied by Hangzhou Saifei technology Co. Ltd. Initially, each membrane sample was pre-compacted (transmembrane pressures (TMP) = 0.15 MPa) with DI water for more than two hours to achieve stabilization, and then pure water permeability (*L*_P_) was measured at 0.06, 0.09 0.12, and 0.15 MPa separately. To minimize investigation errors, five samples were fabricated and measured for each membrane. *L*_P_ was calculated according to Equation (1):

(1) $$L_{P}=\frac{Q}{A\Delta tP}$$

where *Q* represents the volume of permeates (*L*), *A* stands for the effective filtration area (m^2^) and Δ*t* (h) and *P* (MPa) are the time and operation pressure of permeation, respectively.

Afterwards, an HA (5 mg/L, pH = 7.0) or BSA (200 mg/L, pH = 7.4) solution was used instead of DI water to measure the permeation fluxes and rejection ratios of the membranes. This process was operated at 0.10 MPa for 90 min. Permeation flux (*J*) and rejection (*R*) can be calculated according to Equations (2) and (3) [37]:

(2) $$J=\frac{Q}{A\Delta t}$$

(3) $$R=\left(\frac{C_{F}-C_{P}}{C_{F}}\right)\times 100\%$$

where *C*_F_ and *C*_P_ are the solute concentrations in feed and permeate, respectively. The concentrations of HA and BSA solutions were obtained by the peaks of 254 and 280 nm in the UV–VIS spectrum, respectively. The molecular weight cutoff (MWCO) values of various membranes were determined by rejection experiments of various 500 mg/L PEG solutions (i.e., 8, 10, 12, 20, and 35 kDa). The molecular weight of the solute with 90% rejection was defined as the MWCO.

### 2.5. Antifouling Property Evaluation

The antifouling capacity of the membranes was evaluated by three phases and performed in two cycles. Each cycle included a pure water filtration (*J*_w_) measurement for 0.5 h, followed by feed (5 mg/L HA) solution filtration (*J*_P_) for 3 h, and lastly physical washing with DI water for 0.5 h. A fouling resistance model was employed to estimate the flux loss due to fouling. Therefore, the flux recovery ratio (*FRR*), total flux decline ratio (*DR*_t_), reversible flux decline ratio (*DR*_r_), and irreversible flux decline ratio (*DR*_ir_) were analyzed. The *DR*_t_ was calculated according to Equation (4), which can be subdivided into two components *DR*_r_ and *DR*_ir_. *DR*_r_ was calculated according to Equation (5), and it referred to the flux decline that could be easily recovered, which was mainly caused by reversible foulants deposition. Instead, *DR*_ir_ was caused by foulants blocked in the pores of membrane, which was hardly recovered, and was calculated according to Equation (6). In addition, the indexes of the flux recovery ratio (*FRR*) were calculated using Equation (7) [38,39] as follows:

(4) $${DR}_{t}=1-J_{P}/J_{W1}$$

(5) $${DR}_{r}=\left(J_{W2}-J_{P}\right)/J_{W1}$$

(6) $${DR}_{ir}=1-J_{W2}/J_{W1}$$

(7) $$FRR=J_{W2}/J_{W1}$$

where *J*_w1_ and *J*_w2_ are pure water fluxes of the membrane during the 1st- and 2nd-cycle filtration experiments, respectively; *J*_P_ is the steady permeation flux of different formulated membranes during HA filtration.

### 2.6. Antibacterial Activity Evaluation

Sterilization ratios have been always used to qualitatively investigate the antibacterial property of membranes. In the work, *Escherichia coli* (*E. coli*) was used as model strains combined with a colony-forming counting method to measure the quantity of surviving bacteria, which could reflect the real status of bacteria on the membrane surface. The membrane samples (1 cm × 3 cm) were put in cuvettes containing diluted *E. coli* suspension with a predetermined concentration. After cultivation at 37 °C for 24 h, bacteria on the membrane surface were washed carefully by 20 mL normal saline (0.9 wt % NaCl). The collected bacteria suspensions were diluted to a suitable concentration by applying a standard serial dilution method and spread onto an Luria–Bertani (LB) solid culture medium afterwards. The visible bacterial colonies were acquired after the culturing in an incubator at 37 °C for another 24 h. At least six replicates of each sample were measured to allow for statistical evaluation of results. The sterilization ratio (*E*_b_) can be calculated according to Equation (8):

(8) $$E_{b}=\left(\frac{N_{b}-N_{m}}{N_{b}}\right)\times 100\%$$

where *N*_b_ and *N*_m_ represent the numbers of bacterial colonies on the surface of the neat PSf/PES membrane (blank control) and modified hybrid membrane, respectively.

Fluorescence microscopy observation is another simpler and more straightforward approach to investigating the activity of attached bacterial cells on the surfaces of membranes. The contaminated membrane samples that were incubated in *E. coli* suspension were washed with a phosphate-buffered saline (PBS) solution 3 times at 37 °C to remove loosely attached bacteria. Bacteria, which were difficult to remove, were stained with 0.1% acridine orange (dyeing living bacterium) and 10 μg·mL^−1^ propidium iodide (dyeing dead bacterium) and then observed by confocal laser scanning microscopy (CLSM) images at a 10× magnification. During observation, living bacteria appeared green or yellow-green color; meanwhile, dead bacteria showed red color [40,41]. At least 20 images were photographed for each substrate to quantify the bacteria adhesion to the membrane surface.

## 3. Results

### 3.1. Chemical Structures and Compositions of the Membrane Surfaces

**Figure 3:**
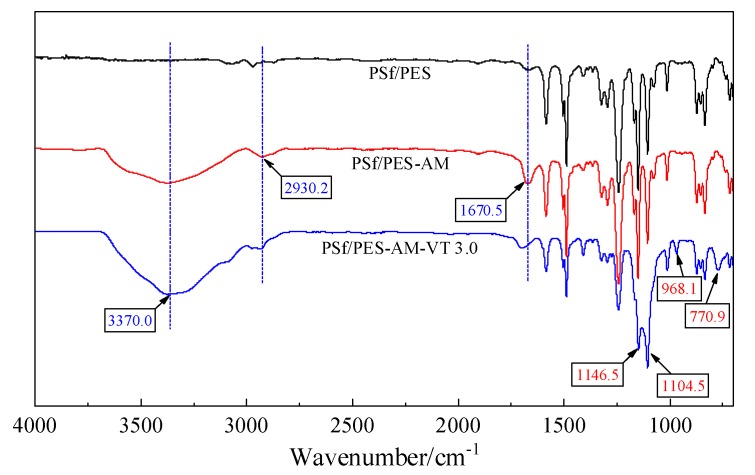
FTIR–ATR spectra of the neat and hybrid polymer membranes.

**Figure 4:**
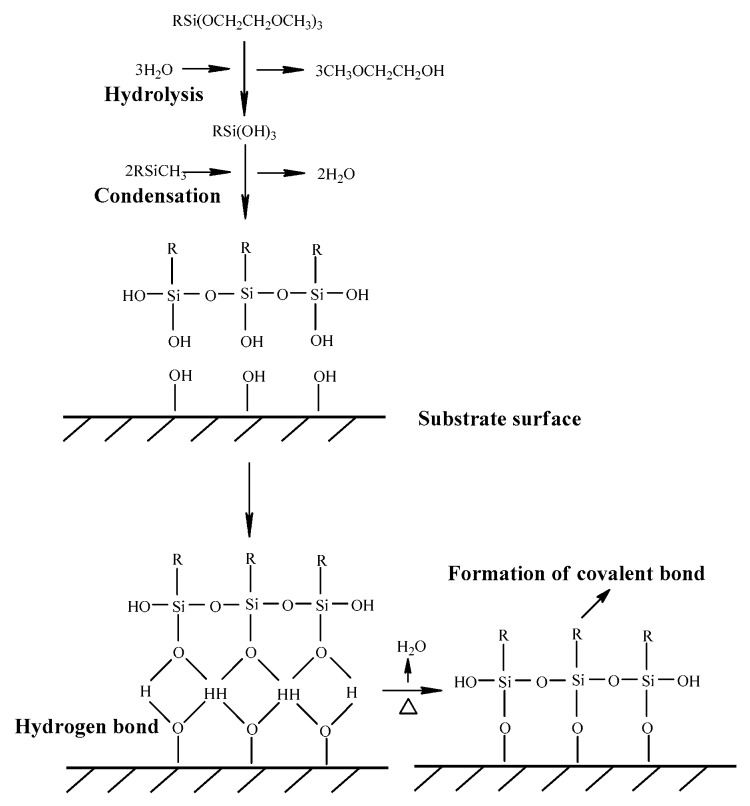
Coupling reaction processes of a silane coupling agent and inorganic materials.

In order to investigate the functional groups present on the hybrid membrane surfaces, FTIR/ATR spectra after each step analysis were measured. The FTIR spectra are depicted in Figure 3, and the neat PSf/PES membrane without modifier was taken as a blank control. It can be seen that several new peaks associated with the C=C stretching vibration at 1695–1630 cm^−1^, –CH_2_– vibrations at 2975–2845 cm^−1^, and O–H stretching vibrations at 3370 cm^−1^ were observed, after the capsaicin derivative AMTHBA was successfully self-polymerized via an “MWA in situ polymerization-blending” process [28]. It is known that silicone molecules can adhere to the hydroxy group on the surface of a substrate to form a network silicone film with excellent mechanical durability and antifouling property (as shown in Figure 4) [33,42,43]. The AMTHBA-incorporated casting solution rich in hydroxyl could provide many reactive sites for silane coupling agents. As expected, for the silane-modified PSf/PES-AM-VT 3.0 membrane, the peaks of a new group Si–O–Si originating from the hydrolysis condensation of VTMES were observed at around 1146, 1105, and 771 cm^−1^ [34]. Therefore, AMTHBA and VTMES can be introduced into the membranes via the novel two-step modification strategy to endow polymeric membranes with dual antifouling and antibacterial properties.

**Figure 5:**
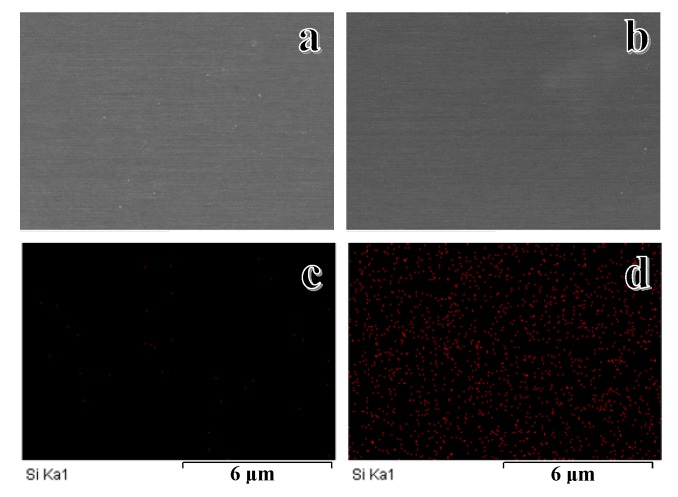
SEM of the PSf/PES-AM membrane (**a**) and the PSf/PES-AM-VT 3.0 membrane (**b**). Silicon distributions of the PSf/PES-AM membrane (**c**) and the PSf/PES-AM-VT 3.0 membrane (**d**).

To further verify the successful incorporation of VTMES in the hybrid polymer membranes, the dispersion of the silicon element within the PSf/PES-AM membrane (Figure 5a) and the PSf/PES-AM-VT 3.0 membrane (Figure 5b) was confirmed by EDS spectra. Obviously, little signals can be seen on the surface of the PSf/PES-AM membrane (Figure 5c), and red light dots, which represent the existence of silicon, were clearly observed on the surface of the modified PSf/PES-AM-VT 3.0 membrane (Figure 5d), demonstrating well-distributed VTMES within the polymer matrix.

### 3.2. Morphologies of the Membranes

**Figure 6:**
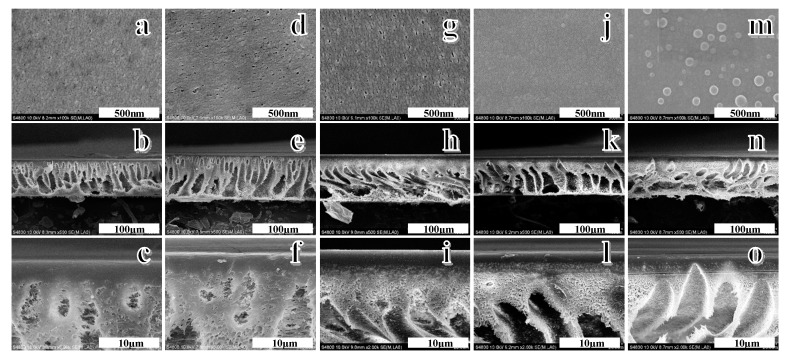
SEM images of the PSf/PES membrane (**a**–**c**), the PSf/PES-AM-VT 0.5 membrane (**d**–**f**), the PSf/PES-AM-VT 1.0 membrane (**g**–**i**), the PSf/PES-AM-VT 2.0 membrane (**j**–**l**), and the PSf/PES-AM-VT 3.0 membrane (**m**–**o**) at different magnifications.

The top-surface and cross-section morphologies of various polymer membranes were characterized by SEM micrographs, and the SEM images are depicted in Figure 6. It can be observed that the surface porosity and pore size of the PSf/PES-AM-VT membrane (Figure 6d,g) obviously increased with the addition of VTMES. This is mainly due to AMTHBA playing a leading role in the co-modification process when a small amount of VTMES was added, which was consistent with the results—the addition of AMTHBA leads to an increase in mean pore size [28]. Subsequently, the hybrid membrane exhibited a much denser surface without an obvious pore structure (Figure 6j), when the content of VTMES was 2 wt %. One possible reason is that the silane coupling agent decreased the interactions between the polymer (PSf/PES) and the solvent (DMAc), resulting in the acceleration of solvent/nonsolvent exchange velocity during the phase inversion process, which formed a compact surface [44]. Interestingly, regular round-shaped indentations occurred on the membrane surface at a VTMES content of 3.0 wt % (Figure 6m), which revealed a flatness and smoothness surface only can be obtained when a small amount of VTMES (<2.0 wt %) was added. For the cross-section images, all the membranes exhibited typical asymmetric porous structures comprised of a dense top layer and a porous supporting layer, indicating that the incorporated functional polymers had less impact on the membrane-forming ability of PSf/PES. However, the porous supporting layer transformed from finger-like pores to double drop-shaped pores (Figure 6n) with the increasing VTMES content, which is the cause of round-shaped indentations formed on the surface. The trend can be explained by the increase of thermodynamic stability of the membrane due to addition of hydrophobic VTMES, which in turn affect the precipitation rate during the phase inversion process.

### 3.3. Surface Wettability of the Membranes

**Figure 7:**
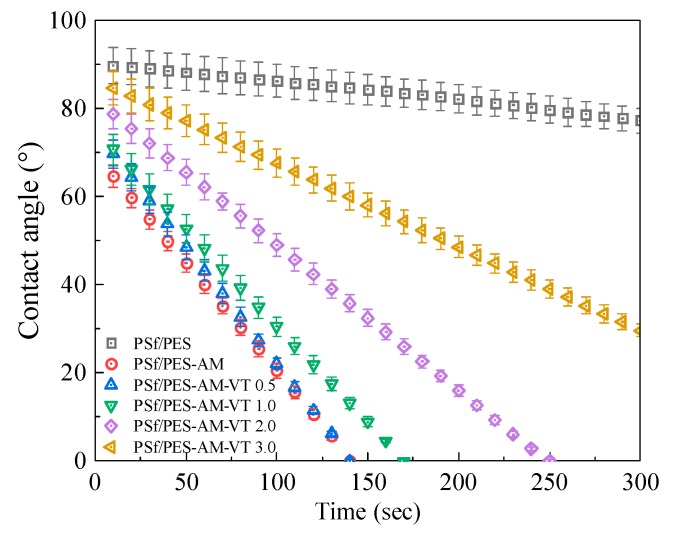
Time-dependent water contact angles of various membranes.

For UF membranes, the surface hydrophilicity and wettability of membranes play crucial roles in both controlling membrane water transport properties and alleviating membrane fouling during separation [45]. Hydrophilicity and wettability can be determined by static and time-dependent WCAs on the membrane surface, respectively, which is a measure of the tendency for water to wet the membrane. Figure 7 illustrates that the PSf/PES-AM membrane had the lowest contact angle among all the membranes, attributed to the inherent abundance of hydroxyl groups in AMTHBA [28]. By increasing the added content of VTMES from 0.5 to 3.0 wt % in hybrid membranes, the WCA increased from 69.6° to 84.7°, indicating attenuation in hydrophilic characteristics of PSf/PES-AM-VT membranes. Unfortunately, the infiltration performances of the hybrid membranes also reduced significantly with the increase of VTMES content. For the case of incorporating a small amount of VTMES in the membrane (0.5 wt %), the time-dependent WCA decreased to zero within 140 s, which is the same with in the PSf/PES-AM membrane. While increasing the VTMES content (3.0 wt %), it can be found that the water droplets hardly permeated through the membrane and the WCA remained at 29.6° after 300 s. The reasons for the impairment may be as follows: (a) a higher concentrcrosslinkation of VTMES in the casting solution could react with more amounts of hydroxy groups, resulting in decrease of hydrophilic groups; (b) the smooth and dense skin layer formed by the crosslinking between hydroxy groups and VTMES made it harder for water molecules to permeate through the silane-modified membrane. Thus, to retain the hydrophilicity and wettability of the PSf/PES-AM-VT membranes, up to 1.0 wt % of VTMES would be an appropriate option.

### 3.4. Mechanical Properties of the Membranes

**Figure 8:**
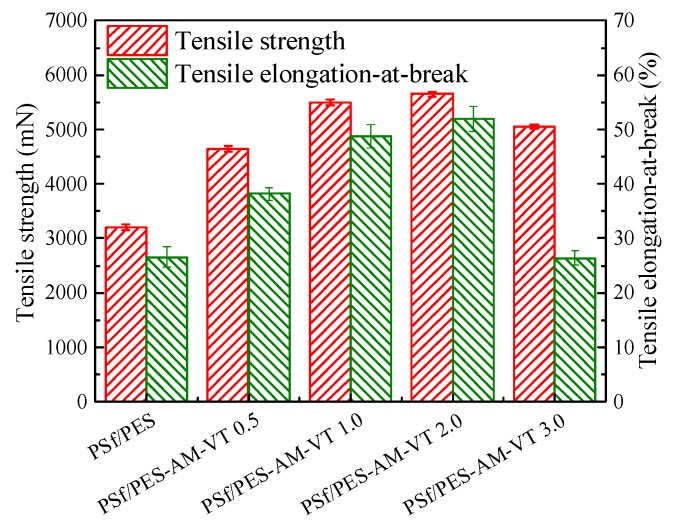
Mechanical properties of the neat and modified membranes.

Mechanical properties are another essential index to estimate comprehensive performances of polymer membranes, which need to sustain pressurized backwash and induced movement (by bubbling, vibrations, etc.) [46]. As depicted in Figure 8, with the enhancement of VTMES content (0.5–2.0 wt %), the mechanical properties of the silane-modified membranes improved and was strongest, when the content of VTMES was 2.0 wt %. The PSf/PES-AM-VT 2.0 membrane was found to have a tensile strength of 5650 mN at an elongation at break of 51.9%. It can be probably ascribed to the crosslinking network between VTMES and the polymer matrices and interfacial interactions via hydrogen bonding, which could restrict the migration of molecular chains, contributing to the improvement of mechanical properties. In addition, it should be noted that a further addition of VTMES (>2.0 wt %) led to a degradation in the overall mechanical strength. This phenomenon may be assigned to the alteration of a membrane structure, i.e., round-shaped indentations on the surface and double drop-shaped pores in the cross-section of the PSf/PES-AM-VT 3.0 membrane acted as mechanically weak, when an external force was imposed on the membrane.

### 3.5. Separation Performance of the Membranes

**Figure 9:**
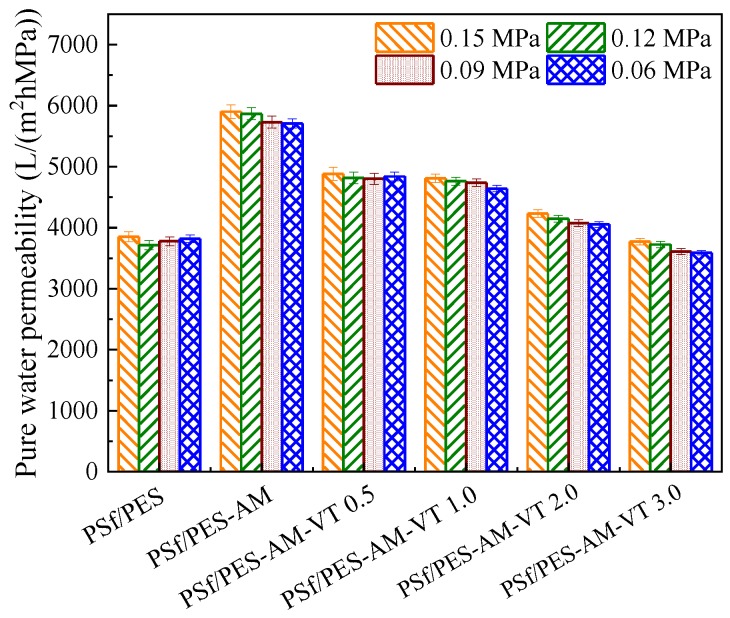
Pure water permeability of various membranes during the pure water filtration.

A permeation test with pure water was carried out to investigate the water transport properties of the dual-functional membranes. As shown in Figure 9, all the membranes exhibited a relative stable pure water permeability (*L*_P_) in the 0.09–0.15 MPa range of TMP. Obviously, AMTHBA had dominating impact on the process of co-modification, when the content of VTMES was less than 1.0 wt %, leading to a higher *L*_P_ value of 4738.0 L·m^−2^·h^−1^/MPa for the PSf/PES-AM-VT 1.0 membrane with an increased rate of 66.1% in comparison to in the pure PSf/PES membrane (~3790.4 L·m^−2^·h^−1^/MPa). This can be attributed to the transformation of porous structures and the amelioration in membrane hydrophilicity and wettability. However, a slight decline appeared, when the content of VTMES was more than 1.0 wt %, which may be ascribed to a reduction in membrane hydrophilicity and alteration of the porous structure caused by the crosslinking between the VTMES and the polymer matrix.

**Figure 10:**
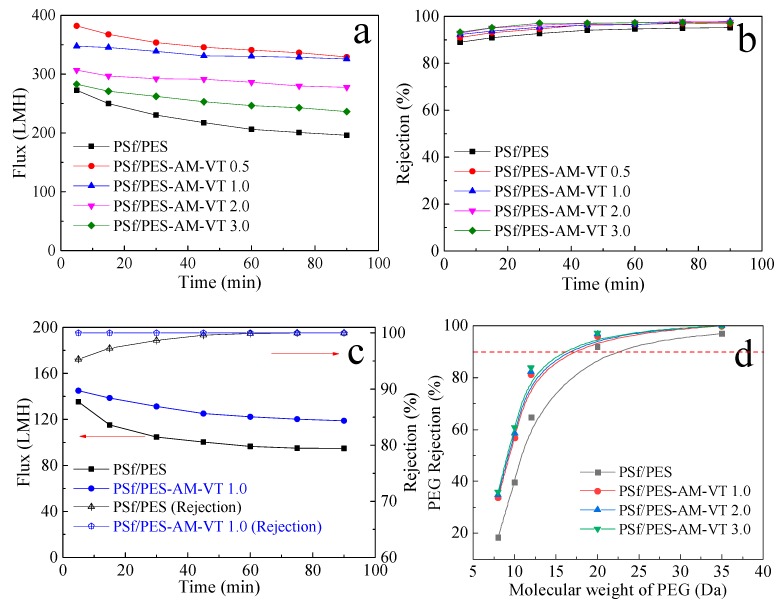
Time-dependent permeate fluxes (**a**) and rejection percentages (**b**) of various membranes during filtration of a 5 mg·L^−1^ humic acid (HA) solution. (**c**) Time-dependent permeate fluxes ) of various membranes during filtration of a 200 mg·L^−1^ bovine serum albumin (BSA) solution. (**d**) PEG rejection as a function of molecular weight cutoff (MWCO) for all the membranes.

Then, an HA solution (5 mg·L^−1^) and a BSA solution (200 mg·L^−1^) were filtrated to evaluate the separation performance of the dual-functional PSf/PES membranes under 0.1 MPa. As presented in Figure 10a, compared with the neat PSf/PES membrane, all the silane-modified membranes exhibited an increased HA permeation flux, which was consistent with *L*_P_. However, the flux decline decreased, and the flux stability improved with the increments in VTMES content. Therefore, although the initial permeation flux for the PSf/PES-AM-VT 1.0 membrane was lower than that for the PSf/PES-AM-VT 0.5 membrane, the steady permeation fluxes of two membranes were approximately equal (~325 L·m^−2^·h^−1^ (LMH)) with an increased rate of 66% in comparison to in the PSf/PES membrane (~196 LMH). Moreover, the rejection of HA was enhanced from 95% for the PSf/PES membrane to 97% for the PSf/PES-AM-VT 3.0 membrane with an increase in VTMES content, as depicted in Figure 10b. These phenomena can be attributed to the relatively dense surface separation layers of the silane-modified membranes. To verify this, the MWCO values of the membranes (as shown in Figure 10d) were further investigated, which is crucial for the separation performance of the composite membranes. The data revealed that the MWCO gradually decreased from 22.7 to 16.0 kDa with an increment of VTMES addition from 0 to 3.0 wt %. By integrating the properties above (i.e., hydrophilicity, wettability, mechanical property, permeability, and separation performance), the PSf/PES-AM-VT 1.0 (with 1.0 wt % VTMES) membrane was applied in the following studies.

Furthermore, the membrane filtration performance of the PSf/PES-AM-VT 1.0 membrane with a BSA solution was investigated, and the result is depicted in Figure 10c. Similarly, the silane-modified membrane exhibited improvement in both permeation flux and BSA rejection, i.e., the flux increased from 94.8 to 118.8 LMH (with a rate increase of 25.3%), and the rejection achieved 100%, breaking the intrinsic “trade-off” characteristic between the permeability and selectivity of polymer membranes.

### 3.6. Antibacterial Activity and Antibacterial Adhesion Ability of the Membranes

**Figure 11:**
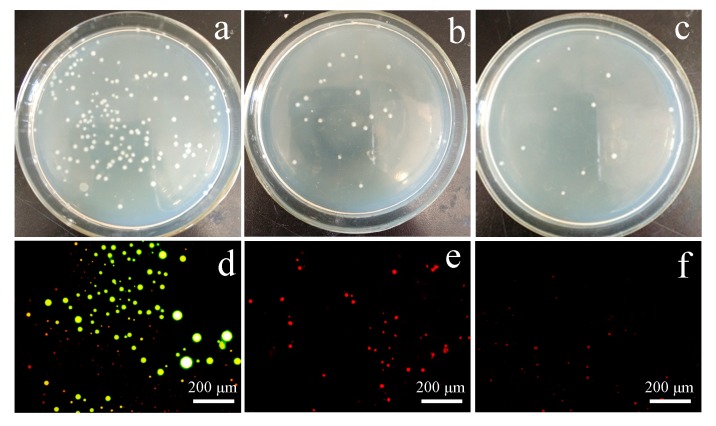
Pictures of Petri dishes for the neat and modified membranes: (**a**) PSf/PES; (**b**) PSf/PES-AM; (**c**) PSf/PES-AM-VT 1.0. Live (in yellow-green)/dead (in red) cell strain analysis of the membranes: (**d**) PSf/PES; (**e**) PSf/PES-AM; (**f**) PSf/PES-AM-VT 1.0.

The antibacterial activities of the dual-functional membranes were quantitatively evaluated by antibacterial efficiency (*E*_b_) towards *E. coli*. The pure PSf/PES membrane was measured as a control. As illustrated in Figure 11a–c, the capsaicin-mimic PSf/PES-AM membrane exhibited excellent an inhibition effect on *E. coli*. Moreover, the bacterial inhibition ability of the PSf/PES-AM-VT 1.0 membrane improved obviously after incorporating the VTMES, and the corresponding *E*_b_ approximately rose from 85.2% to 92.3%, relative to the control. The results revealed that the crosslinking reaction between hydroxyl groups and silicone molecules had absolutely no influence on the antibacterial activities of the capsaicin-mimic material AMTHBA.

The silane-modified PSf/PES-AM-VT membranes were designed to realize antifouling property without sacrificing antibacterial activities, so as to utilize the effective combination of the advantages of both a capsaicin-mimic material AMTHBA (antibacterial) and a silane coupling agent VTMES (antifouling). Thus, the antibacterial adhesion ability of the co-cultured membrane surface was also detected, after it contacted with *E. coli* for 24 h by fluorescence microscopy. As shown in Figure 11d,e, numerous viable bacteria adhered to the pure PSf/PES membrane [28]; while obvious inactivation of bacteria can be seen on the PSf/PES-AM and PSf/PES-AM-VT membranes. The results further confirmed that AMTHBA was still effective against the bacteria. Compared with in the PSf/PES-AM membrane (Figure 11e), the number of deactivated bacteria that adhered to the PSf/PES-AM-VT membrane (Figure 11f) significantly decreased. It demonstrated that the incorporation of a minuscule amount of VTMES in a membrane can create a smooth surface and reduce the amounts of attached bacterial cells, keeping the membrane surface clear.

### 3.7. Antifouling Performances of the Membranes

**Figure 12:**
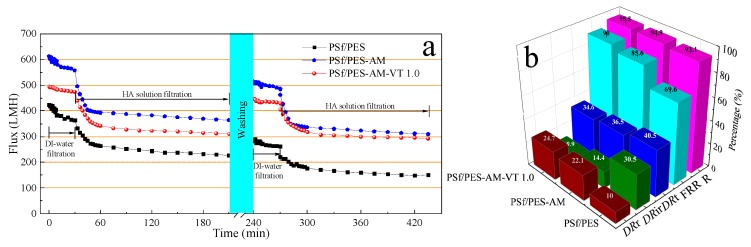
(**a**) Time-dependent flux in two cycles of the resulting membranes in the 5 mg·L^−1^ HA solution filtration process. (**b**) Corresponding reversible flux decline ratio (*DR*_r_), irreversible flux decline ratio (*DR*_ir_), total flux decline ratio (*DR*_t_), flux recovery ratio (*FRR*), and HA rejection (*R*).

To identify the antifouling properties of the dual-functional membranes, cycle-dependent dynamic filtration tests for the PSf/PES, PSf/PES-AM, and PSf/PES-AM-VT membranes were carried out. As depicted in Figure 12a, the fluxes of all the membranes declined precipitously, when water was replaced with an HA solution, and then the permeation fluxes declined slowly and seemed to be stabilized after an operation time of 30 min, which was caused by the absorption and deposition of the fouling layer during the filtration. In the first-cycle filtration, the steady-state permeation fluxes of PSf/PES, PSf/PES-AM, and PSf/PES-AM-VT membrane were found to be 226.7, 364.3, and 311.0 LMH, respectively, while those in the second-cycle filtration were 150.1, 309.3, and 292.5 LMH after physical hydraulic washing for 30 min. Obviously, the gap between the fluxes of the PSf/PES-AM and PSf/PES-AM-VT 1.0 membranes narrowed from 55.0 to 18.5 LMH, demonstrating that the incorporation of VTMES can retard the flux decline because of its excellent antifouling capability.

Figure 12b presents the antifouling properties related to *DR*_r_, *DR*_ir_, *DR*_t_—the sum of *DR*_r_ and *DR*_ir_, *FRR*, and HA rejection (*R*) of the resulting membranes. Obviously, the *FRR* value of the pristine PSf/PES membrane is the lowest, which indicates that serious fouling occurred on the neat membrane surface after filtering the HA solution. The *FRR* value of the PSf/PES-AM membrane increases to 85.6% and then further increases to 90.0% with the addition of 1.0 wt % VTMES to the casting solution, demonstrating an excellent antifouling performance. Generally, a higher *FRR* corresponding to a lower *R*_ir_ value is indicative of higher antifouling performance [47,48]. The *R*_ir_ value of the PSf/PES membrane is 30.5%, whereas this parameter decreases significantly to 9.9% for the PSf/PES-AM-VT 1.0 membrane. It is mainly attributed to the fact that the presence of a network silicone film formed by silicone molecules adhering to hydroxy groups can effectively inhibit the foulants deposition on the membrane surface or foulants blockage in membrane pores. In addition, the PSf/PES-AM-VT 1.0 membrane had an *R* value of over 95%, which slightly varied after the two-cycle pressure filtration, compared with the result shown in Figure 10b. Therefore, the silane-modified membrane was more permeable without sacrificing rejection, as well as displaying improved antifouling performance.

**Figure 13:**
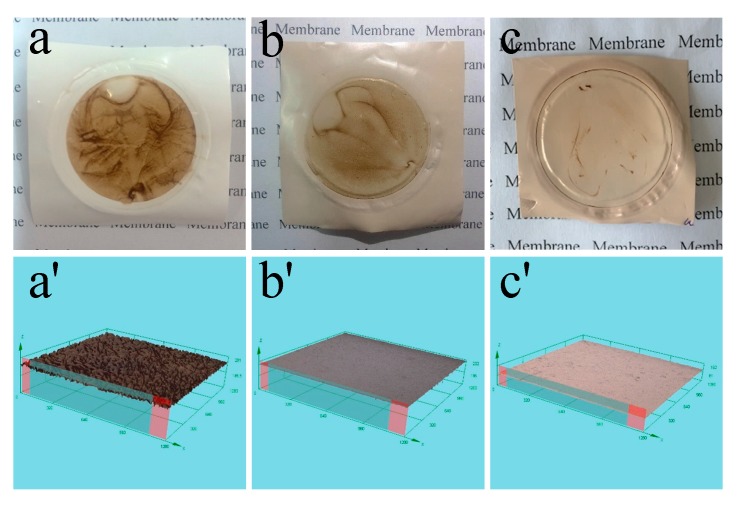
Digital photographs of the PSf/PES (**a**), PSf/PES-AM (**b**), and PSf/PES-AM-VT 1.0 (**c**) membranes surfaces after two cycles of HA solution filtration. Confocal laser scanning microscopy images of the PSf/PES (**a’**), PSf/PES-AM (**b’**), and PSf/PES-AM-VT 1.0 (**c’**) membranes surfaces after two cycles of HA solution filtration

To make the adsorption and deposition of the foulant onto the membrane surfaces more visual, morphological analysis of the fouled membrane surfaces was also done by a digital camera and CLSM. As depicted in Figure 13, the neat PSf/PES (Figure 13a) and PSf/PES-AM membranes (Figure 13b) suffered varying degrees of contamination, while the PSf/PES-AM-VT 1.0 membrane (Figure 13c) was still as clean as new. Likewise, vertical needle-like protuberances under the microscope spread across the PSf/PES membrane surface (Figure 13a’). The silane-modified dual-functional membrane was smooth as new, indicating that the network silicone film on the surface benefited the foulant repellence. In summary, incorporation of a small amount of VTMES only could increase the membrane antifouling performance, and a dual-functional membrane was successfully fabricated.

### 3.8. Comparison with other UF Membranes Reported in Recent Years

Table 2 summarizes the detailed comparative analyses (e.g., antifouling, antibacterial, permeability, and separation performances) of PSf/PES-AM-VT membranes developed in the present work and some PSf/PES (or capsaicin-incorporated) membranes. By comparison, the permeability of PSf/PES-AM-VT membranes is among the best, while their BSA Rejection is the highest. In addition, the antifouling and antibacterial properties of PSf/PES-AM-VT membranes are closed to and even more superior to those of other membranes reported in recent years.

## 4. Conclusions

In the present study, a simple two-step blending strategy was used to switch an intrinsically inert and hydrophobic membrane to a dual-functional (antifouling and antibacterial) and hydrophilic membrane. The versatile dual-functional hybrid membranes were fabricated via the effective combination of the advantages of a capsaicin-mimic material AMTHBA and a silane coupling agent VTMES. The successful preparation of PSf/PES-AM-VT membranes was confirmed by FTIR/ATR, SEM, and EDS mapping. Contact angle measurements demonstrated that the hydrophilicity and wettability of the PSf/PES-AM-VT 1.0 membrane could remain intact, even though partial hydroxyl groups crosslinked with VTMES. Therefore, the resulting polymer membranes exhibited an anti-trade-off characteristic between the permeability and selectivity, i.e., a steady-state HA permeation flux of 326 LMH and a rejection percentage of 97%. Meanwhile, silane-modified membranes containing small amounts of VTMES displayed superior antifouling and antibacterial properties compared with pristine PSf/PES membranes. Taken together, the synthesized dual-functional PSf/PES-AM-VT membrane shows potential to be utilized in water-treatment-related applications.

## Figures and Tables

**Table 1:** Compositions of casting solutions used to prepare the modified membranes.

Membrane	PSf (wt %)	PES (wt %)	DMAc (wt %)	PEG-400 (wt %)	H_2_O (wt %)	AMTHBA (wt %)	AIBN (wt %)	VTMES (wt %)
PSf/PES	12.8	3.2	75.6	8	0.4	0	0	0
PSf/PES-AM	12.8	3.2	73.4	8	0.4	2	0.2	0
PSf/PES-AM-VT 0.5	12.8	3.2	72.9	8	0.4	2	0.2	0.5
PSf/PES-AM-VT 1.0	12.8	3.2	72.4	8	0.4	2	0.2	1
PSf/PES-AM-VT 2.0	12.8	3.2	71.4	8	0.4	2	0.2	2
PSf/PES-AM-VT 3.0	12.8	3.2	70.4	8	0.4	2	0.2	3

**Table 2:** Comparison of PSf/PES-AM-VT membranes developed in the present study with other membranes.

Matrix Membrane	Monomer	Pure Water Flux (L·m^−2^·h^−1^)	BSA Rejection (%)	*FRR* (%)	*E*_b_ (%)	Year of Publication and Reference
PSf/PES	AMTHBA/VTMES	473.8	~100	90	92.3	This work
PSf/PES	AMTHBA	586.8	~100	85.6	--	2018 [28]
PVDF	PMMA-PACMO-capsaicin	225.5	64.6	~90	88.5	2019 [49]
PSf	Aniline	396.1	~98	66.5	--	2019 [50]
PES	Graphene oxide/arabic gum	~520	~79	~75	--	2019 [51]
PSf	Basil seed gum (BSG)	155.3	~84	~100	--	2019 [52]
